# Risks According to the Timing and Frequency of Hypotension Episodes in Postanoxic Comatose Patients

**DOI:** 10.3390/jcm9092750

**Published:** 2020-08-25

**Authors:** Yong Hwan Kim, Jae Hoon Lee, Jung In Seo, Dong Hoon Lee, Won Young Kim, Byung Kook Lee

**Affiliations:** 1 Department of Emergency Medicine, Samsung Changwon Hospital, Sungkyunkwan University School of Medicine, Gyeongsangnam-do 51353, Korea; suka1212@hanmail.net; 2 Department of Emergency Medicine, Dong-A University College of Medicine, Busan 49201, Korea; 3 Division of Convergence Education, Halla University, Wonju 26404, Korea; leehoo1928@gmail.com; 4 Department of Emergency Medicine, Chonnam National University Medical School, Gwangju 35015, Korea; ggodhkekf@hanmail.net (D.H.L.); bbukkuk@hanmail.net (B.K.L.); 5 Department of Emergency Medicine, Ulsan University College of Medicine, Seoul 44033, Korea; wonpia73@naver.com

**Keywords:** out-of-hospital cardiac arrest, hypothermia, induced, hypotension, shock, hemodynamics

## Abstract

The aim of this study was to assess the risk of unfavorable outcomes according to the timing of hypotension episodes in cardiac arrest patients. This prospectively conducted multicenter observational study included 1373 out-of-hospital cardiac arrest patients treated with 33 °C targeted temperature management (TTM). Unfavorable neurological outcome and the incidence of complications were analyzed according to the timing of hypotension. Compared with hypotension before TTM initiation (adjusted hazard ratio (aHR) 1.51), hypotension within 6 h after TTM initiation was associated with an increased risk of unfavorable neurologic outcome (aHR 1.693), and after 24 h of TTM, was connected with decreased risk (aHR 1.277). The risk of unfavorable neurological outcome was gradually reduced over time after TTM initiation. Hypotension, persisting both before and during TTM, demonstrated a greater risk (aHR 2) than transient hypotension (aHR 1.265). Hypotension was correlated with various complications. Differences in lactate levels were persistent, regardless of the initial fluid therapy (*p* < 0.001). Hypotension showed a strong correlation with unfavorable neurological outcome, especially in the early phase after TTM initiation, and complications. It is essential to manage hypotension that occurs at the beginning of TTM initiation to recover cerebral function in cardiac arrest patients.

## 1. Introduction

Low stroke volume and systolic and diastolic dysfunction after cardiac arrest can often result from myocardial stunning [1], a reversible myocardial dysfunction that is observed within the first 24 h after cardiac arrest. This condition usually takes two to three days to recover, but mortality due to persisting hypotension in non-recovering patients tends to be high [1,2]. It is also known that vasodilation can occur within up to 72 h [2]. Postresuscitation myocardial dysfunction and vasodilation can induce hypotension episodes, which, in turn, threaten the survival of patients. Furthermore, many adverse effects that have been associated with targeted temperature management (TTM), such as pneumonia or sepsis, acute kidney injury, arrhythmia, and hypoglycemia or hyperglycemia [3,4,5,6,7] could contribute to cardiovascular dysfunction.

Myocardial dysfunction and vasodilatation due to myocardial stunning, hypovolemia, sepsis, etc., can cause hypotension in post-cardiac arrest patients. For management of these causes, several treatments have been used. Postresuscitation myocardial dysfunction requires inotropic support, and norepinephrine, and fluids are known as the most effective treatments for vasodilation [8]. Correcting hypotension (systolic blood pressure (SBP) less than 90 mmHg) may be reasonable [9]. Infusion of relatively large volumes of fluid is endurable in patients with cardiac arrest [8].

In a recent study on patients with hypotension episodes after cardiac arrest, it was found that the survival rate of patients undergoing 36 °C TTM was not significantly different from that of patients undergoing 33 °C TTM, and those who underwent 36 °C TTM had an even lower incidence of adverse events [10]. However, there is still controversy in terms of whether hypotension at the timing during 33 °C TTM is associated with unfavorable neurological outcome [11].

There is no doubt that hypotension is a significant risk factor in patients with cardiac arrest [12]. Many studies have reported that hypotension episodes found within a specific time window after restoration of spontaneous circulation (ROSC) are associated with unfavorable neurological outcome [13,14,15,16], but the alteration of prognoses according to the occurrence of hypotension at various time points during the whole period from admission to discharge (approximately one week if there were no severe complications) has not been examined. Therefore, this study aimed to determine how hypotension episodes, according to the timing of hypotension before TTM initiation and during the TTM period, are correlated with unfavorable neurological outcome or the incidence of complications.

## 2. Materials and Methods

### 2.1. Study Design and Setting

This study is a prospectively conducted multicenter observational cohort study based on the Korean Hypothermia Network prospective registry (KORHN-PRO). The KORHN, a multicenter clinical research consortium for TTM in Korea, collected data from out-of-hospital cardiac arrest (OHCA) patients treated with TTM in advanced critical care settings at 22 teaching hospitals throughout South Korea. This study was approved by the institutional review board of all participating hospitals and registered at the International Clinical Trials Registry Platform (NCT02827422). The data were obtained with informed consent and recorded in a web-based data registration system in accordance with the Utstein-style guidelines [17] and were reviewed by the investigator and clinical research coordinator of each site. Two data managers and three clinical research associates in the main research center regularly monitored and reviewed the quality of the data, with feedback from the investigator of the corresponding site. The 22 centers implemented TTM according to the 2015 American Heart Association guidelines and used their own protocols with detailed instructions when there was no suggestion in the guidelines (management of vasoactive drugs and fluids, rewarming time, etc.). The protocol of the present study was shared in advance so that the required variables could be obtained. The study design and plan, including the informed consent form, were approved by the institutional review board of each hospital. In accordance with national requirements and the principles of the Declaration of Helsinki, written informed consent was obtained from all patients’ legal surrogates.

### 2.2. Study Population

**Figure 1:**
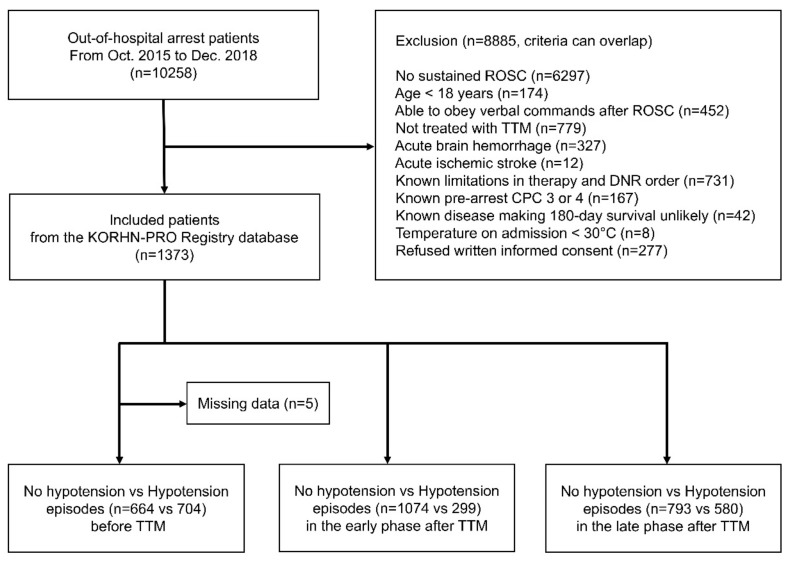
Screening and distribution of the patients. TTM, targeted temperature management; ROSC, restoration of spontaneous circulation; CPC, cerebral performance category; DNR: do not resuscitate; KORHN-PRO, Korean Hypothermia Network prospective registry.

Among 10,258 cardiac arrest patients enrolled between October 2015 and December 2018, 1373 comatose patients who were treated with mild therapeutic hypothermia after OHCA were included to investigate how unfavorable neurological outcome and adverse effects change according to the time window of hypotension. The inclusion criteria were as follows: patients over 18 years old, patients with OHCA regardless of underlying cause, patients with an unconscious mental status (Glasgow Coma Scale score < 8) after ROSC, and patients treated with 33 °C TTM. The exclusion criteria were as follows: patients who had rearrest events or death within 24 h on admission, acute hemorrhagic or ischemic stroke (according to a local protocol for stroke patients, they were treated with prolonged TTM for about 7 days), limitations in therapy or a do not resuscitate (DNR) order, a prearrest cerebral performance category (CPC) score of 3 or 4, chronic diseases yielding little probability of survival over 180 days, a body temperature of <30 °C on admission; and patients whose caregiver did not sign the written informed consent form were excluded from the study (Figure 1). The patients were divided into the no-hypotension and hypotension groups before TTM initiation and in the early and late phase after TTM initiation on the basis of the hypotension episodes’ timing at admission or in the early and late phase that were previously studied (Figure 1) [10,18]. Additionally, the no-hypotension and hypotension groups were compared to identify the relation with unfavorable neurological outcome and complications before TTM initiation and during the TTM period.

### 2.3. Variables and Endpoints

**Figure 2:**
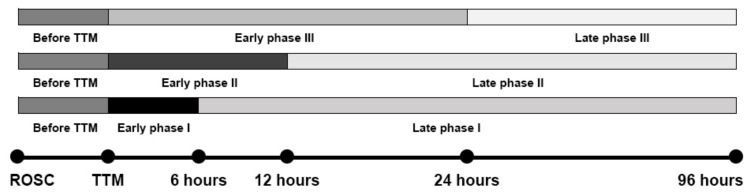
Time frame for the timing of hypotension episodes after out-of-hospital cardiac arrest.

All blood pressures were obtained from an arterial line or a noninvasive blood pressure cuff for 4 days. If the hourly blood pressure had at least one episode of hypotension within a 6-hour period, the lowest value was recorded and considered to be a hypotension episode. Hypotension episodes were defined as systolic blood pressure <90 mmHg for >30 min or requiring supportive measures to maintain a blood pressure of 90 mmHg [9,10]. Differences between the two groups, i.e., patients with and without hypotension occurring before TTM (from ROSC to TTM initiation), were investigated first. Moreover, hypotension episodes during the TTM period were divided into those in the early and late phases after TTM initiation. The main analysis of unfavorable neurological outcome was conducted by comparing patients with hypotension episodes occurring within 6 h and after 6 h, within 12 h and after 12 h, and within 24 h and after 24 h of starting TTM (the early phase was defined as the time window from TTM initiation to 6 (Early phase I), or 12 (Early phase II,) or 24 h (Early phase III) after the initiation, and the late phase was defined as the time window after 6 (Late phase I) or 12 (Late phase II) or 24 h (Late phase III) of TTM) (Figure 2). Additionally, patients with hypotension episodes occurring and persisting before TTM initiation and during the TTM period (persistent hypotension group) were also compared with patients with hypotension occurring either before TTM initiation or during the TTM period regardless of whether the hypotension episodes were in the early or late phase after TTM initiation (transient hypotension group) and patients with no hypotension.

Basic demographic variables, resuscitation variables, and postresuscitation variables were obtained. The basic demographic variables that were investigated included age, sex, body mass index, and past medical history. The resuscitation variables of interest were witnessed arrest, bystander CPR, initial prehospital electrocardiographic rhythm, absolute anoxic time (from arrest to the CPR initiation), relative anoxic time (from CPR initiation to ROSC), cardiac arrest cause, percutaneous coronary intervention, and the four-scale score on admission (eye, motor, brainstem, and respiration response). The main postresuscitation variables related to hypotension episodes that were evaluated, included the lactate level measured initially and at 6-hour intervals; daily balance of fluid input and output (insensible water loss was calculated to be 500 mL); target body temperature; duration of TTM (TTM duration may be prolonged if rebound hyperthermia occurs); length of intensive care unit (ICU) stay; cardiovascular sequential organ failure assessment (SOFA) score; and use of vasoactive or inotropic agents, such as dopamine, norepinephrine, vasopressin, epinephrine, and dobutamine. Fluid balance and lactate levels were analyzed to determine hypotension management and its effect.

The frequency of complications found within 7 days after ROSC was evaluated between the no-hypotension group and the hypotension group before TTM initiation. The following complications due to cardiac arrest were included: seizure; significant retroperitoneal, muscular, solid organ, or thoracic organ bleeding with a decrease in hemoglobin >5 g/dL and >2 units of transfused blood as well as symptomatic bleeding in a critical organ; infection, including severe sepsis or septic shock; hypokalemia (<3 mEq/L); hypophosphatemia (<2.2 mg/dL); hypomagnesemia (<1.7 mg/dL); hypoglycemia (blood glucose <60 mg/dL); sustained hyperglycemia (blood glucose >180 mg/dL for >4 h); tachycardia (>130 beats/min) requiring control of heart rate; bradycardia (<40 beats/min); and rearrest.

The primary outcome of this study was the difference in unfavorable neurological outcome, defined as a CPC score of 3–5 at 6 months, according to the timing and frequency of hypotension episodes in patients treated with 33 °C TTM. The second outcome was whether significant differences in the incidence of complications were shown between the hypotension groups before TTM initiation. Furthermore, the prolongation of the length of ICU stay due to hypotension was examined to infer the recovery time of surviving patients, excluding patients with sudden death.

### 2.4. Statistical Analysis

A sample size of 307–520 patients was calculated at a two-sided alpha level of 0.05, with approximately 90% power, based on previous studies that showed a 65–73% mortality rate in patients with hypotension and a 44–53% mortality rate in patients with no hypotension on admission [10,19]. Considering the large number of independent variables and missing data for each independent variable, more subjects than the calculated sample size were included in the present study.

Essential variables related to hypotension episodes, such as the 6-hour interval of lactate and the daily balance of input and output, were analyzed to compare differences among the hypotension and no-hypotension groups using a generalized linear mixed model with repeated measurements during the TTM period. The model included adjustment for baseline values for all variables. Kaplan–Meier plots were used with a log rank test and Cox regression was performed to analyze the relationships among hypotension episodes according to a special time window and unfavorable neurological outcome, making allowance for significant covariates. All tests were two sided, and a *p*-value of less than 0.05 was considered to indicate significance.

## 3. Results

**Figure 3:**
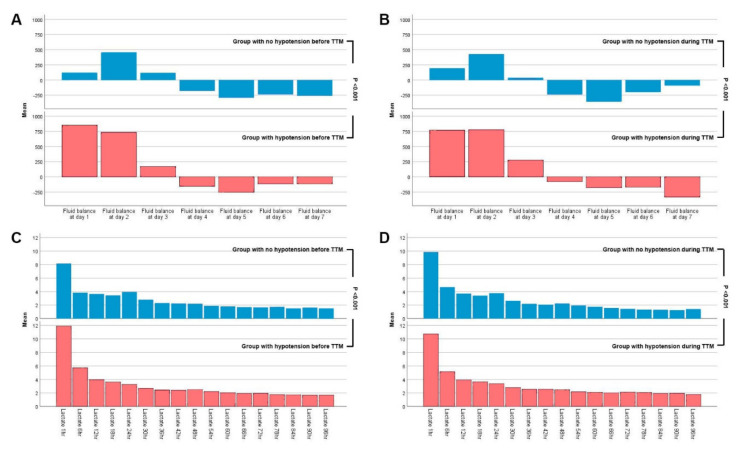
Fluid balance and lactate level. Changes in fluid balance according to admission date compared between the no-hypotension group and the hypotension group before TTM initiation (**A**) and during the TTM period (**B**). Changes in lactate level over time compared between the no-hypotension group and the hypotension group before TTM initiation (**C**) and during the TTM period (**D**). The lactate level and fluid balance after 2 days showed statistically significant differences between the two groups.

Of 1373 patients assessed for eligibility, 664 patients were assigned to the group with no hypotension, and 704 patients were assigned to the group with hypotension. The patients were further divided according to hypotension episodes before TTM initiation as follows: 1074 and 299 patients in the early phase within 12 h after TTM initiation, respectively, and 793 and 580 patients in the late phase after 24 h of TTM, respectively (Figure 1). The difference in baseline characteristics was compared in patients at admission. Patients with hypotension found before TTM initiation were shown to be older in age and to have a longer relative anoxic time, more frequent occurrences of an asystole pattern, and less frequent occurrences of ventricular fibrillation in initial electrocardiography, lower four-scale scores, slightly higher target temperatures, and longer TTM durations than those with no hypotension found before TTM initiation (Table 1). Hypotension episodes before TTM initiation were associated with a higher risk of unfavorable neurological outcome (57.3% vs. 79.5%, *p* < 0.001), higher cardiovascular SOFA scores (4 vs. 3 points, *p* < 0.001), initial lactate levels (10.9 mg/dL vs. 8.5 mg/dL, *p* < 0.001), and creatinine levels (1.4 mg/dL vs. 1.2 mg/dL, *p* < 0.001), and greater fluid imbalance (935 mL vs. 46 mL, *p* < 0.001) (Table 1). Cardiovascular SOFA scores showed a tendency to decrease over time (4 points at Days 1 and 2; 3 points at Day 3; 2 points at Day 4; 1 point at Day 5; and zero at Days 6 and 7). Larger amounts of dopamine and norepinephrine were administered in the hypotension group before TTM initiation (Table 1). Lactate levels and fluid balance, which were repeatedly measured over time, demonstrated statistically significant differences, irrespective of massive hydration infusion, due to the distinctive changes in lactate levels and fluid balance from TTM initiation to Day 2 (*p* < 0.001 and *p* < 0.001) (Figure 3).

**Figure 4:**
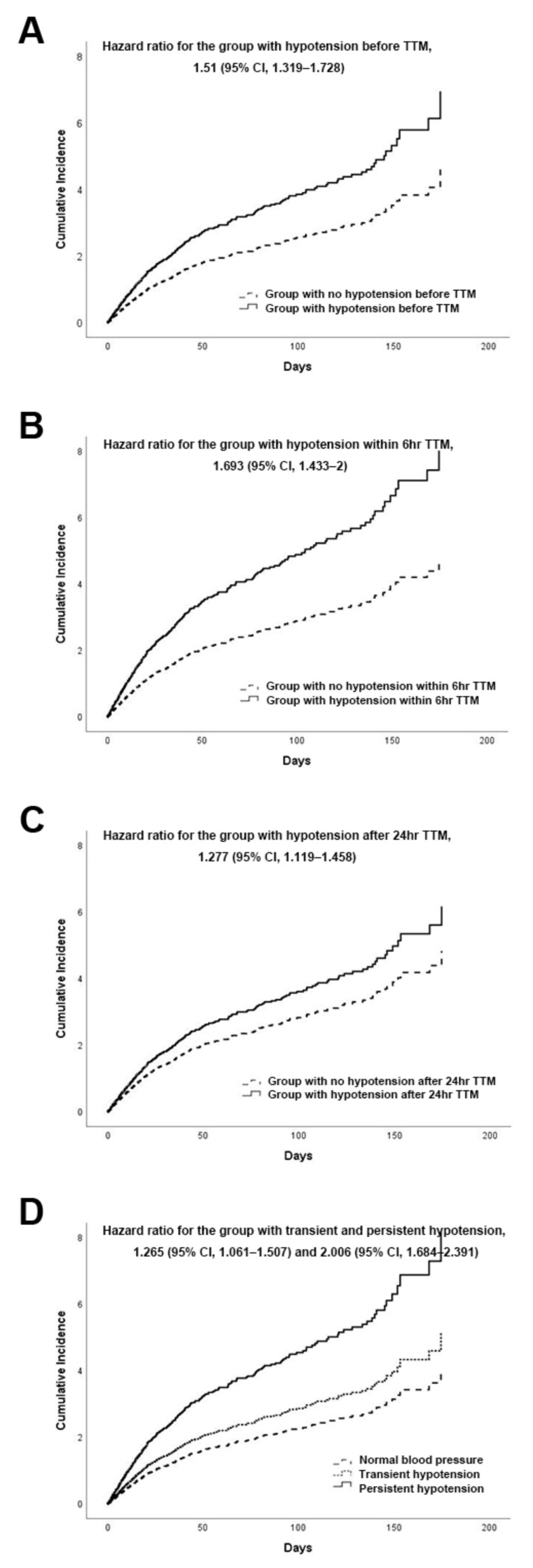
The cumulative risks of unfavorable neurological outcome according to the timing of hypotension episodes. Cumulative incidence curves for the hypotension group before TTM (**A**), the hypotension group within 6 h after TTM (**B**), the hypotension group 24 h after TTM (**C**), and the group with hypotension episodes both before TTM initiation and during the TTM period (**D**) as compared with those for the normal blood pressure group.

Favorable neurological outcome manifested as CPC scores of one and two at day 180 were found in 20.5% of the 704 patients with hypotension before TTM initiation and in 20.7% of the 701 patients with hypotension during the TTM period. Compared with the no-hypotension group, the groups with hypotension found before TTM initiation and during the whole TTM period showed the same hazard ratio ((HR) 1.51, *p* < 0.001). No significant differences were shown in the distribution of CPC scores between the two groups, but the early (within 6 (Early phase I) or 12 (Early phase II) or 24 h (Early phase III) after TTM initiation) and late phase (after 6 (Late phase I) or 12 (Late phase II) or 24 h (Late phase III) of TTM) hypotension groups (Figure 2) showed the following significant differences in prognoses according to the HRs based on the timing of hypotension episodes during the TTM period: HR, 1.693 with *p* < 0.001 within 6 h after TTM initiation and HR, 1.428 with *p* < 0.001 after 6 h of TTM; HR, 1.662 with *p* < 0.001 in the hypotension group within 12 h after TTM initiation and HR, 1.353 with *p* < 0.001 after 12 h of TTM; and HR, 1.527 with *p* < 0.001 within 24 h after TTM initiation and HR, 1.277 with *p* < 0.001 after 24 h of TTM. The risks of unfavorable neurological outcome due to hypotension episodes were increased in the early phase after TTM initiation and were alleviated in the late phase after TTM initiation. Figure 4B,C indicates a representative risk in the early and late phases after TTM initiation, respectively. Moreover, the patients were further divided into the following three groups: patients with normal blood pressure both before TTM initiation and during the TTM period, patients with transient hypotension episodes either before TTM initiation or during the TTM period, and patients with persistent hypotension episodes both before TTM initiation and during the TTM period. It was also identified that the HRs in the hypotension groups tended to gradually increase according to the frequency of hypotension episodes (HR 1.265 and *p* = 0.009 in the transient group with hypotension either before TTM initiation or during the TTM period; HR 2.006 and *p* < 0.001 in the persistent group with hypotension both before TTM initiation and during the TTM period) as compared with the group that maintained normal blood pressure (Figure 4D). The analytical results for unfavorable neurological outcome were adjusted by statistically significant covariates, including age, witnessed arrest, time from CPR initiation to ROSC, four-scale score, target temperature, TTM duration, and the presence of a shockable rhythm.

Complications such as severe sepsis or septic shock, acute renal failure requiring renal replacement therapy, hypoglycemia or hyperglycemia, tachycardia, and rearrest were observed most frequently in the hypotension group before TTM initiation (Table 2). However, compared with those in the group with hypotension before TTM initiation, the group with hypotension during the TTM period did not have an increased incidence of complications (significant infection, 12.4% vs. 13.4%, *p* = 0.324; acute renal failure requiring renal replacement therapy, 24.7% vs. 22.8%, *p* = 0.246; hypoglycemia, 14.9% vs. 15.5%, *p* = 0.659; hyperglycemia, 58.8% vs. 60.6%, *p* = 0.393; tachycardia, 7.6% vs. 8%, *p* = 0.678; bradycardia, 8.9% vs. 7.4%, *p* = 0.174; rearrest, 27.8% vs. 25.2%, *p* = 0.057, respectively). Furthermore, the length of ICU stay of the surviving patients was not different between the hypotension and no-hypotension groups before TTM initiation.

Lactate levels and fluid balance in the hypotension group increased for the first two days, but there was a statistically significant difference in the lactate level between the no-hypotension group and the hypotension group after two days (*p* < 0.001) that was irrespective of massive hydration in the early phase.

## 4. Discussion

### 4.1. The Overall Results

In this study, the rates of unfavorable neurologic outcome were significantly different among patients suffering from episodes of hypotension and those without such episodes before TTM initiation or during TTM, and hypotension episodes were associated with various complications. It is paramount that the risk of unfavorable neurological outcome was gradually reduced over time after TTM (HR 1.693, 1.662, and 1.572 in the early phase within 6, 12, and 24 h after TTM initiation; and 1.428, 1.353, and 1.277 in the late phase after 6, 12, and 24 h of TTM to Day 4, respectively). We identified that hypotension episodes were most hazardous in the early phase after TTM initiation, then the phase before TTM initiation showed a lesser HR for hypotension episodes, and the least hazardous phase was the late phase after TTM initiation. This fact has never been reported before. These findings suggest that those occurring hypotension episodes could be the crucial risk factor for unfavorable neurological outcome or various complications, and hemodynamic management in the early phase after TTM initiation could be essential.

### 4.2. Hypotension Episodes and Unfavorable Neurological Outcome in Cardiac Arrest Patients

It has been demonstrated that hemodynamic instability is found in more than half of cardiac arrest patients within 7 h after ROSC [2], and 73% of patients experience hypotension within 24 h after ROSC [20]. Hypotension was related to a worsening patient prognosis, and many studies have been conducted to determine the effects of hypotension episodes at a specific point in time and reported that SBP <90 mmHg at admission or 1 h or 6 h or 24 h after admission was associated with unfavorable neurological outcome [13,14,20,21]. Consistent with findings from a previous study that more than two episodes of SBP <100 mmHg within 6 h after the ROSC were associated with unfavorable neurological outcome [21], our study also showed that patients with hypotension episodes both before TTM initiation and during the TTM period had the poorest neurological outcome.

### 4.3. Hypotension Episodes in the Early Phase after Targeted Temperature Management (TTM) Initiation

It is understandable that repeated hypotension is associated with poorer neurological outcome, but it is notable that hypotension episodes within 6 h or 12 h after TTM initiation were found to be comparatively more hazardous. In a previous study on hypotension episodes found within 6 h and 72 h after TTM initiation in pediatric patients with cardiac arrest, it was shown that patients with hypotension episodes in the early phase after TTM initiation (within 6 h after TTM initiation) had a poorer neurological outcome than those with hypotension episodes within 72 h after TTM initiation [18]. Consistent results were shown in the present study, which included adult patients (within 6 h vs. within 4 days after TTM initiation: HR 1.693 vs. 1.51). Hemodynamic degradation in the early phase after TTM initiation was also observed in another study including adult patients. Patients with unfavorable neurological outcome were shown to have a lower SBP within 6 h after TTM initiation and lower mean arterial pressure within 12 h after TTM initiation than those with a favorable neurological outcome [22].

The fact that hypotension episodes in the early phase after TTM initiation are most dangerous can be attributed to the fact that the brain or heart are especially vulnerable to hypotension in the early phase after TTM initiation [18] or to the fact that TTM performed within 24 h after ROSC decreases brain blood flow, such as the mean flow velocity in the middle cerebral artery, resulting in more damage to the brain [23,24]. Moreover, cerebral autoregulation can be reduced or right shifted after OHCA [25]. Therefore, it is very important to maintain the blood pressure of the patient in the early phase after TTM initiation.

In a randomized controlled trial including cardiac arrest patients with hypotension, it was found that these previously reported positive and negative effects on the heart and blood vessels did not yield any differences in survival rates as comparing with patients treated with 33 °C TTM and those treated with 36 °C TTM [10]. However, significant increases in ICU mortality (but not 180-day mortality), lactate levels, and cardiovascular SOFA scores were observed in patients treated with 33 °C TTM. Only patients with SBP less than 90 mmHg at admission were defined as cardiogenic shock patients in that study [10]. Although cardiac arrest patients could experience hypotension before TTM initiation and during the TTM period, the effects of TTM according to the timing of hypotension were not considered. Hypotension at admission (before TTM initiation) was less dangerous than hypotension in the early phase after TTM initiation, and hemodynamic instability in the early phase after TTM initiation due to TTM or underlying disease should be considered for the adjudication of TTM effectiveness.

### 4.4. Vasoactive Agents and Fluids

Hemodynamic instability in cardiac arrest patients leads to the administration of vasoactive agents and substantial fluid management. Norepinephrine-induced increases in perfusion pressure and cardiac index have been associated with unfavorable neurological outcome following OHCA [26]. Moreover, in one study, the use of both dopamine and norepinephrine or epinephrine in cardiac arrest patients has resulted in poorer neurological outcome than the use of dopamine alone [27], and various vasoactive agents were connected with mortality in another study [28]. In the present study, norepinephrine and dopamine, which were the main vasoactive agents used as the first-line treatment, were administered more frequently in the hypotension group. These differences could be because a larger amount of vasoactive agents cause unfavorable neurological outcome and also because hypotension episodes and poorer patient condition lead to frequent use of these agents. In addition, several studies have reported that lactate levels were increased in the early phase after TTM initiation but normalized over time and did not affect the prognosis of the patients [12,29]. Unfavorable neurological outcome and lactate levels were not improved, although larger amounts of vasoactive agents and fluids were infused to patients in hypotension groups. Hypotensive patients with higher lactate levels who were receiving higher doses of vasoactive agents could undergo improper blood pressure control or receive no treatment apposite for underlying causes of cardiac arrest.

### 4.5. Complications Due to Hypotension Episodes and TTM

Complications due to hypotension episodes after TTM initiation are another consideration. TTM is known to increase the risk of pneumonia and sepsis [3]. In our study, it was also shown that hypotension episodes were associated with a higher incidence of severe sepsis and septic shock, but there was no increase in the incidence of severe infection (excluding mild infection) during the TTM period (13.4% before TTM initiation vs. 12.4% during the TTM period). TTM could induce mild infections, such as focal pneumonia, due to decreased immunity but did not increase the incidence of fatal infections. Some previous studies have reported that both hyperglycemia and hypoglycemia were associated with unfavorable neurological outcome [4,5]. Certainly, in our study, the incidences of hypoglycemia and hyperglycemia were increased in hypotensive patients. A heart rate <60 beats/min or sinus bradycardia <50 beats/min have been known to indicate favorable neurological outcome in postanoxic patients treated with TTM [30,31], while tachycardia has been known to indicate unfavorable neurological outcome [6]. Nonetheless, in our study, the incidence of bradycardia less than 40 beats/min was slightly increased in hypotensive patients before TTM initiation. A moderate decrease in heart rate of between 40 and 60 beats/min could have a positive effect on prognosis or hypotension [30]. Bradycardia less than 40 beats/min might not be related to favorable neurological outcome if the bradycardia is not sinus bradycardia. Acute kidney injury is a well-known indicator for poor prognosis in patients after cardiac arrest [7], and the incidence of severe renal failure that required renal replacement therapy was increased in our hypotensive patients. As shown in Table 2, these complications were common in the hypotension groups, but the incidences of complications were similar between the hypotension groups before TTM initiation and during the TTM period. It was also revealed that the length of ICU stay was not significantly longer in the no-hypotension group among surviving patients. These results indicate that hypotension episodes are associated with many complications, but the frequency of complications is not likely to show a difference between the phases before and after TTM initiation.

### 4.6. Limitations

The limitations of this study are as follows: First, patients included in this study were not randomized according to the presence of hypotension. Second, some significant data, including mean arterial pressure, stroke volume, and brain blood flow, are missing. Hemodynamic monitoring can be important in that it may guide proper management according to volume status and cardiac function in postanoxic patients. Since this was a multicenter study, the assessment of fluid responsiveness or hemodynamic monitoring may have been applied differently, and it cannot be assured that each hospital properly administered fluid and vasopressors or inotropes. Third, the causes of cardiac arrest or hypotension were not fully evaluated. Causes of cardiac arrest were not identified in approximately 15% of the included patients. Proper management according to the cause of cardiac arrest or hypotension could improve neurological outcome of the patients.

## 5. Conclusions

Hypotension episodes before TTM initiation and during the TTM period are closely associated with unfavorable neurological outcome and complications such as significant infection, renal failure, hypoglycemia, hyperglycemia, tachycardia, bradycardia, and rearrest. Persistent hypotension denoted a greater risk of unfavorable neurological outcome than transient hypotension. Most importantly, compared with hypotension episodes before TTM initiation and in the late phase after TTM initiation, hypotension episodes in the earlier phase after TTM initiation were more hazardous and showed a tendency of decreasing HRs over time after TTM.

The patients with hypotensive episodes showed higher mortality and lactate levels, although more fluids were administered to the patients in the beginning. Therefore, it is essential to use extra caution in cardiac arrest patients with frequent hypotension episodes who are undergoing TTM, especially in the early stage after TTM initiation, to assess the causes of hypotension, and to carry out intensive hemodynamic management.

## Figures and Tables

**Table 1:** Baseline characteristics.

Variable	All Patients (*n* = 1368)	No Hypotension Before TTM (*n* = 664)	Hypotension Before TTM (*n* = 704)	*p*-Value ^5^
Age, years	62 (51, 74)	61 (49, 72)	64 (53, 76)	<0.001
Male, *n* (%)	974 (71.2)	484 (72.9)	490 (69.6)	0.189
BMI, kg/m^2^	23.4 (20.9, 25.7)	23.4 (21.1, 25.7)	23.3 (20.8, 25.4)	0.163
Systolic blood pressure, mmHg	120 (90, 154)	130 (104,161)	110 (80, 147)	<0.001
Witness arrest, *n* (%)	946 (69.2)	489 (74.5)	457 (65.6)	<0.001
Bystander CPR, *n* (%)	840 (61.4)	406 (62.2)	434 (62.4)	0.955
Time from arrest to CPR initiation, minutes	1 (0, 7)	1 (0, 7)	1 (0, 7)	0.685
Time from CPR initiation to ROSC, minutes	25 (15, 38)	22 (12, 34)	30 (18, 43)	<0.001
Time from ROSC to TTM initiation, hours	3.4 (2.2, 4.9)	3.4 (2.1, 4.9)	3.4 (2.2, 4.8)	0.53
Initial prehospital ECG rhythm				<0.001
Asystole, *n* (%)	444 (32.5)	185 (30.8)	259 (43.1)	
PEA, *n* (%)	268 (19.6)	127 (21.1)	141 (23.5)	
Pulseless VT, *n* (%)	15 (1.1)	10 (1.7)	5 (0.8)	
VF, *n* (%)	448 (32.7)	270 (44.9)	178 (29.6)	
Already achieved ROSC at EMS arrival, *n* (%)	27 (2)	9 (1.5)	18 (3)	
Previous history				
Cardiovascular disease ^1^, *n* (%)	285 (20.8)	145 (21.8)	140 (19.9)	0.375
Neurologic disease ^2^, *n* (%)	137 (10)	60 (9)	77 (10.9)	0.242
Pulmonary disease, *n* (%)	106 (7.7)	37 (5.6)	69 (9.8)	0.003
Malignancy, *n* (%)	80 (5.8)	42 (6.3)	38 (5.4)	0.465
Psychologic disease, *n* (%)	50 (3.7)	23 (3.5)	27 (3.8)	0.714
Cardiac cause	850 (62.1)	431 (81.6)	419 (78.5)	0.219
Causes of cardiac arrest				0.13
Medical, *n* (%)	851 (62.2)	432 (65.1)	419 (59.5)	
Trauma, *n* (%)	28 (2)	14 (2.1)	14 (2)	
Submersion, *n* (%)	19 (1.4)	5 (0.8)	14 (2)	
Electrocution, *n* (%)	6 (0.4)	3 (0.5)	3 (0.4)	
Drug overdose, *n* (%)	15 (1.1)	5 (0.8)	10 (1.4)	
Asphyxia, *n* (%)	77 (0.56)	29 (4.4)	48 (6.8)	
Hanging, *n* (%)	160 (11.7)	79 (11.9)	81 (11.5)	
Others, *n* (%)	112 (8.2)	97 (14.6)	115 (16.3)	
PCI, *n* (%)	207 (15.1)	112 (41)	95 (41.7)	0.478
Four score ^3^	0 (0, 3)	1 (0, 4)	0 (0, 2)	<0.001
Cardiovascular SOFA ^4^ at day 1	4 (2, 4)	3 (0, 4)	4 (4, 4)	<0.001
Total dose of dopamine, µg/kg	5605 (1651, 21,600)	3553 (1104, 19,200)	6489 (1834, 22,941)	0.012
Total dose of norepinephrine, µg/kg	108 (30, 360)	50 (18, 182.4)	162 (48, 576)	<0.001
Total dose of vasopressin, IU/min	31 (7, 113)	31 (7, 118)	32 (7.2, 108)	0.919
Total dose of epinephrine, µg/kg	68 (18, 271)	57 (16, 145)	70 (18.3, 308)	0.566
Total dose of dobutamine, µg/kg	3600 (800, 15,969)	3480 (959, 12,789)	3996 (719, 16,709)	0.966
Input/output at day 1, mL	453 (−354, 1725)	46 (−647, 1078)	935 (−37, 2341)	<0.001
Initial lactate, mg/dL	9.7 (6.1, 12.9)	8.5 (4.8, 11.5)	10.9 (7.5, 14.1)	<0.001
Initial creatinine, mg/dL	1.3 (1.1, 1.8)	1.2 (1, 1.6)	1.4 (1.1, 2.1)	<0.001
Target temperature, °C	33 (33, 34)	33 (33, 33)	33 (33, 35)	<0.001
Duration of TTM, hours	24 (24, 24)	24 (24, 24)	24 (24, 24)	0.018
CPC 3–5, *n* (%)	940 (68.8)	380 (57.3)	560 (79.5)	<0.001

Values are expressed as a number (%) or median (interquartile range). BMI, body mass index; CPR, cardiopulmonary resuscitation; ROSC: restoration of spontaneous circulation; TTM: targeted temperature management; EMS, emergency medical service; PEA, pulseless electric activity; VT, ventricular tachycardia; VF, ventricular fibrillation; PCI, percutaneous coronary intervention; SOFA, sequential organ failure assessment. ^1^ Cardiovascular disease included diseases such as cardiac arrest, coronary artery disease, and congestive heart failure. ^2^ Neurological diseases included diseases such as transient ischemic accidents, stroke, and other neurological diseases. ^3^ The four-scale score consisted of eye response, motor response, brainstem reflexes, and respiration and ranged from 0 to 4. ^4^ Cardiovascular SOFA scores ranged from 0 to 4 (0, no hypotension; 1, MAP <70 mmHg; 2, dopamine ≤5 µg/kg/min or dobutamine; 3, dopamine >5 µg/kg/min or epinephrine ≤0.1 µg/kg/min or norepinephrine ≤0.1 µg/kg/min; 4, dopamine >15 µg/kg/min or epinephrine >0.1 µg/kg/min or norepinephrine >0.1 µg/kg/min). ^5^ The *p*-value was calculated by means of Fisher’s exact test and the Mann–Whitney U test.

**Table 2:** Comparison of complications and the length of ICU stay according to the timing of hypotension episodes.

Variable	All Patients (*n* = 1368)	No Hypotension Before TTM (*n* = 664)	Hypotension Before TTM (*n* = 704)	*p*-Value ^5^
Seizure, *n* (%)	325 (23.8)	144 (21.7)	181 (25.7)	0.077
Significant bleeding, *n* (%)	65 (4.8)	25 (3.8)	40 (5.7)	0.115
Significant infection ^1^, *n* (%)	117 (8.6)	23 (3.5)	94 (13.4)	<0.001
RRT ^2^, *n* (%)	248 (18.1)	89 (13.4)	159 (22.8)	<0.001
Hypokalemia, *n* (%)	448 (32.7)	206 (31.1)	242 (34.5)	0.173
Hypophosphatemia, *n* (%)	433 (31.7)	205 (31.2)	228 (33.1)	0.467
Hypomagnesemia, *n* (%)	368 (26.9)	177 (27.2)	191 (27.9)	0.816
Hypoglycemia, *n* (%)	153 (11.2)	45 (6.8)	108 (15.5)	<0.001
Hyperglycemia, *n* (%)	706 (51.6)	283 (42.8)	423 (60.6)	<0.001
Tachycardia ^3^, *n* (%)	79 (5.8)	27 (4.1)	56 (8)	0.003
Bradycardia ^4^, *n* (%)	96 (7)	44 (6.7)	52 (7.4)	0.614
Rearrest, *n* (%)	259 (18.9)	82 (12.3)	177 (25.2)	<0.001
ICU length of stay in survivors, days	34.1 (16.9, 50.5)	34.9 (17.9, 50.9)	32.5 (15.3, 50.2)	0.133

^1^ Significant infection denotes severe sepsis or septic shock. ^2^ RRT denotes renal replacement therapy for the treatment of acute renal failure. ^3^ Tachycardia greater than 130 beats/min requiring treatment was regarded as a complication. ^4^ Bradycardia less than 40 beats/min was regarded as a complication. ^5^ The relationship between hypotension and complications were adjusted making allowance for significant covariates using the Cochran–Mantel–Haenzel Test.
